# An efficient and reliable DNA-based sex identification method for archaeological Pacific salmonid (*Oncorhynchus* spp.) remains

**DOI:** 10.1371/journal.pone.0193212

**Published:** 2018-03-14

**Authors:** Thomas C. A. Royle, Dionne Sakhrani, Camilla F. Speller, Virginia L. Butler, Robert H. Devlin, Aubrey Cannon, Dongya Y. Yang

**Affiliations:** 1 Ancient DNA Laboratory, Department of Archaeology, Simon Fraser University, Burnaby, British Columbia, Canada; 2 Fisheries and Oceans Canada, West Vancouver, British Columbia, Canada; 3 BioArCh, Department of Archaeology, University of York, York, United Kingdom; 4 Department of Anthropology, Portland State University, Portland, Oregon, United States of America; 5 Department of Anthropology, McMaster University, Hamilton, Ontario, Canada; University of Florence, ITALY

## Abstract

Pacific salmonid (*Oncorhynchus* spp.) remains are routinely recovered from archaeological sites in northwestern North America but typically lack sexually dimorphic features, precluding the sex identification of these remains through morphological approaches. Consequently, little is known about the deep history of the sex-selective salmonid fishing strategies practiced by some of the region’s Indigenous peoples. Here, we present a DNA-based method for the sex identification of archaeological Pacific salmonid remains that integrates two PCR assays that each co-amplify fragments of the *sexually dimorphic on the Y chromosome* (*sdY*) gene and an internal positive control (*Clock1a* or D-loop). The first assay co-amplifies a 95 bp fragment of *sdY* and a 108 bp fragment of the autosomal *Clock1a* gene, whereas the second assay co-amplifies the same *sdY* fragment and a 249 bp fragment of the mitochondrial D-loop region. This method’s reliability, sensitivity, and efficiency, were evaluated by applying it to 72 modern Pacific salmonids from five species and 75 archaeological remains from six Pacific salmonids. The sex identities assigned to each of the modern samples were concordant with their known phenotypic sex, highlighting the method’s reliability. Applications of the method to dilutions of modern DNA samples indicate it can correctly identify the sex of samples with as little as ~39 pg of total genomic DNA. The successful sex identification of 70 of the 75 (93%) archaeological samples further demonstrates the method’s sensitivity. The method’s reliance on two co-amplifications that preferentially amplify *sdY* helps validate the sex identities assigned to samples and reduce erroneous identifications caused by allelic dropout and contamination. Furthermore, by sequencing the D-loop fragment used as a positive control, species-level and sex identifications can be simultaneously assigned to samples. Overall, our results indicate the DNA-based method reported in this study is a sensitive and reliable sex identification method for ancient salmonid remains.

## Introduction

Pacific salmonids (*Oncorhynchus* spp.) were and continue to be an important component of many Indigenous fisheries in northwestern North America [1,2]. Ethnographic records indicate many Indigenous salmonid fisheries in the region likely employed sex-selective fishing strategies [3–10]. Among some groups, such as the Tlingit [7], sex-selective fishing was one of the resource management strategies used to cultivate salmonid stocks [11]. Documenting the deep history of these ethnographically-documented sex-selective salmonid fishing strategies and their use as a resource management strategy requires the accurate sex identification of archaeological salmonid remains. Unfortunately, archaeological salmonid bones are frequently fragmented and typically lack sexually dimorphic features, precluding the sex identification of these remains using conventional morphological approaches. Since sex among many salmonids is believed to be primarily genetically determined [12], ancient DNA (aDNA) analysis can potentially be used to identify the sex of archaeological salmonid bones.

Sex is determined in fish through a variety of behavioural, environmental, and genetic mechanisms [13]. Among salmonids, sex is thought to be primarily determined through a genetic system in which males are the heterogametic sex (XY chromosomal sex-determination system) [12]. For many years, the gene responsible for sex differentiation among salmonids was unknown [12]. However, recent studies suggest the *sexually dimorphic on the Y chromosome* (*sdY*) gene is likely the master sex-determining gene in many salmonids, including Pacific salmonids [14–17]. An early study found that among rainbow/steelhead trout (*Oncorhynchus mykiss*), the expression of *sdY*, which is limited to the testis and peaks during testis differentiation, is linked to the development of testis [17]. *sdY*’s role in sex determination is further supported by its presence in the vast majority of male Pacific salmonids from all four tested species (cherry [*O*. *masou*], Chinook [*O*. *tshawytscha*], sockeye [*O*. *nerka*], and rainbow/steelhead trout) and absence in most females [14–17]. Since *sdY* is likely the genus’s master sex-determining gene, all male Pacific salmonids can be expected to carry the gene, which can be detected through a PCR assay [17]. In such an assay, the absence of *sdY* amplicons is indicative of a female, while the presence of *sdY* amplicons is indicative of a male [17].

In this study, we developed and optimized a DNA-based method for the sex identification of archaeological Pacific salmonid remains that incorporates two PCR assays that co-amplify *sdY* and an internal positive control (IPC). In the first assay (*Clock1a*/*sdY*), a short 95 bp fragment of *sdY* is co-amplified alongside a 108 bp fragment of the autosomal *Clock1a* gene, which serves as an IPC. The second assay (D-loop/*sdY*) co-amplifies the same 95 bp fragment of *sdY* and an IPC consisting of 249 bp fragment of the mitochondrial D-loop region. Based on the results of the two assays, a final consensus sex identity can be assigned to a sample. We evaluated the reliability of this method by comparing the known phenotypic sex of 72 modern Pacific salmonids from five species and the sex identities assigned to them with this method. We subsequently tested the method’s sensitivity by applying it to dilutions of modern salmonid DNA and 75 salmonid remains from six species that were recovered from archaeological sites in northwestern North America. Our results indicate the proposed DNA-based method is a highly sensitive and reliable sex identification method for archaeological salmonid remains.

## Materials and methods

### Development of sex identification method for degraded DNA samples

Using an alignment of published and unpublished *sdY* sequences, we designed ten primer pairs that targeted a ~100 bp fragment of *sdY*. The efficiency and specificity of the primer pairs was evaluated through the software NetPrimer (http://www.premierbiosoft.com/netprimer) and/or by testing them on 6 modern salmonid samples (4 males, 2 females). Based on these results, we selected a single primer pair consisting of primers *sdY*-F19 and *sdY*-R20 to include in two PCR assays (Table 1). These assays were designed to co-amplify the 95 bp fragment of *sdY* targeted by this primer pair and an IPC. The IPC acts as a proxy for the X chromosome, which was not directly targeted due to a lack of data regarding X-linked markers conserved across Pacific salmonids. The IPC is also used to assess whether the failure to amplify *sdY* is due to its biological absence or a lack of amplifiable template DNA [18].

**Table 1:** Primers included in the PCR assays used in this study.

Locus	Primer	Sequence (5’-3’)	Amplicon Size
*Clock1a*	*Clk1a*-F50 (F)^1^	TAGCCATGTCTGTGTGTTTACTTGC	108 bp
	*Clk1a*-R60 (R)^1^	GCAGCCAGCTAATTKGATTTG	
D-loop	Smc7 (F)^2^	AACCCCTAAACCAGGAAGTCTCAA	249 bp
	Smc8 (R)^2^	CGTCTTAACAGCTTCAGTGTTATGCT	
*sdY*	*sdY*-F19 (F)	CCCAACACCCTTCCTATCTCC	95 bp
	*sdY*-R20 (R)	CCTTCCTCCCTAGAGCTTAAAAC	

^1^F indicates a forward primer and R denotes a reverse primer.

^2^ Previously published primers from Yang et al. [19].

In the first assay (*Clock1a*/*sdY*), primers *sdY*-F19 and *sdY*-R20 are included in a co-amplification that amplifies the targeted 95 bp *sdY* fragment alongside a 108 bp fragment of the autosomal *Clock1a* gene. This *Clock1a* fragment was targeted with primers *Clk1a*-F50 and *Clk1a*-R60 (Table 1) and serves as an IPC. The second assay (D-loop/*sdY*), which also includes primers *sdY*-F19 and *sdY*-R20, co-amplifies the same *sdY* fragment and an IPC consisting of a 249 bp fragment of the mitochondrial D-loop region. This D-loop fragment was amplified with primers (Smc7 and Smc8) previously published in Yang et al. [19] (Table 1). Following Speller and Yang [18], we set up the *Clock1a*/*sdY* and D-loop/*sdY* co-amplifications to preferentially amplify *sdY* by targeting IPCs longer than the *sdY* fragment and weighting the primer ratios in favour of the *sdY* primers. A primer ratio of 1.5:1 (*sdY*:*Clock1a* primers) was used in the *Clock1a*/*sdY* assay while a primer ratio of 6:1 (*sdY*:D-loop primers) was used in D-loop/*sdY* assay.

### Modern salmonid samples

To evaluate the reliability of our proposed sex identification method, we applied it to 72 modern Pacific salmonids of known phenotypic sex. These modern samples consisted of: tissue samples obtained from pre-deceased salmonids purchased at a public market in Steveston, BC, from a Fisheries and Oceans Canada (DFO) licensed commercial sockeye salmon fisher operating in Barkley Sound, BC; muscle and skin tissue collected from carcasses of pre-deceased spawned-out salmonids washed up on the banks of the Coquitlam River (Port Coquitlam, BC); archived DNA samples held at Simon Fraser University (Burnaby, BC); archived tissue samples provided by DFO (West Vancouver, BC); and tissue samples collected from live salmonids reared by DFO. The live reared salmonids used in this study were reared and collected in compliance with the Canadian Council for Animal Care guidelines under permit 15-001R1 issued by DFO’s Pacific Regional Animal Care Committee. The live salmonids were reared at DFO’s Centre for Aquaculture and Environmental Research (West Vancouver, BC) in a dryland facility designed to prevent the escape of cultured salmonids. Prior to obtaining tissue samples, the live reared salmonids were euthanized in a bath of tricaine methanesulfonate (MS-222; 100 mg/L) buffered with sodium bicarbonate (200 mg/L). Tissue samples were obtained from the sacrificed salmonids after all ventilation activity had ceased. No other permits were required for this study. The analyzed modern salmonid samples include males and females from five Pacific salmonid species (Table 2 and S1 Table). DNA was extracted from the modern samples using a DNeasy Blood and Tissue Kit (Qiagen, Valencia, CA) following the manufacturer’s protocols. All pre-PCR laboratory work involving modern salmonid samples was conducted at the Centre for Forensic Research, Simon Fraser University, in a DNA laboratory dedicated to modern samples.

**Table 2:** Species and sex distribution of the modern Pacific salmonid samples.

Species	Males	Females	Total
Chinook	10	10	20
Chum	6	5	11
Coho	10	10	20
Pink	7	3	10
Sockeye	6	5	11
**Total**	39	33	72

### Dilution series

The concentration of DNA in a modern female (KCH4) and male (KCH9) Chinook salmon sample was quantified in triplicate using a NanoDrop 2000c spectrophotometer (Thermo Fisher Scientific, Waltham, MA). The concentration of total genomic DNA in these two modern samples was 1,560.1 ± 1.87 ng/μL (KCH4) and 1,575.8 ± 7.96 ng/μL (KCH9) (mean ± SD). Subsequently, we serially diluted KCH4 and KCH9 10-fold to 1:1,000,000 with distilled H_2_O. To test the sensitivity of our sex identification method, we applied the *Clock1a*/*sdY* and D-loop/*sdY* assays to each of the six dilutions (1:10, 1:100, 1:1,000, 1:10,000, 1:100,000, and 1:1,000,000 dilutions) in the KCH4 and KCH9 dilution series. For both assays, the PCR reaction volumes used for each of the dilutions of KCH4 and KCH9 contained 2.5 μL of DNA solution. Based on their initial concentrations and amount of DNA solution used, the PCR reaction volumes used for the dilutions included approximately 390,025 to 3.9 pg (KCH4) and 393,950 to 3.9 pg (KCH9) of total genomic DNA, respectively.

### Archaeological salmonid remains

To further assess its sensitivity, we applied our sex identification method to archaeological Pacific salmonid remains recovered from three archaeological sites located in British Columbia and Oregon (Table 3). Kawumkan Springs Midden (KSM) (35KL9-12) is a residential village located along the Sprague River in the Upper Klamath Basin, Oregon [20,21]. The salmonid remains from Kawumkan Springs Midden we analyzed are approximately 5,300 to 1,200 years old or are of unknown age [20,21]. Keatley Creek (EeRl-7) is a winter pithouse village in the Interior Plateau region of British Columbia and is situated on a river terrace along the east bank of mid-Fraser River [22]. The Keatley Creek salmonid remains examined in this study were recovered from Late Plateau to Early Kamloops Horizon (~1,500 to 1,100 years BP) living floors and storage pits from three residential structures (Housepits 3, 12, and 107) and one specialized structure (Housepit 9) [23]. Say-Umiton (DhHr-18) is a permanent residential site located in a cove along the southwestern shore of Indian Arm, British Columbia [24]. In this study, we included salmonid remains recovered from Late Phase (~1,200 to 250 years BP) activities areas at Say-Umiton [24].

**Table 3:** Species distribution of the archaeological Pacific salmonid samples.

Site	Site Number	State/Province	Age of Samples (years BP)	Chinook	Coho	Chum	Pink	Rainbow/Steelhead Trout	Sockeye	Total
Kawumkan Springs Midden	35KL9-12	OR	5,300–1,200 /Unknown	2	0	0	0	7	0	9
Keatley Creek	EeRl-7	BC	1,500–1,100	8	2	0	0	0	45	55
Say-Umiton	DhHr-18	BC	1,200–250	0	0	9	2	0	0	11
**Total**				10	2	9	2	7	45	75

In total, 75 archaeological salmonid remains from these three sites were selected for analysis (Table 3). These archaeological samples were selected for sex identification because of their availability, species diversity, and good mtDNA preservation. Mitochondrial DNA has been amplified from all 75 of these samples during previous projects [20,23,25,26]. DNA was originally extracted from these samples using a modified silica-spin column method [27,28]. All of the samples were previously identified to the species-level through the analysis of cytochrome *b* and/or D-loop fragments [20,23,25,26]. In total, six species of Pacific salmonids are represented among the analyzed assemblages (Table 3 and S2 Table). All pre-PCR laboratory work involving the archaeological samples was conducted in a dedicated ancient DNA laboratory in the Department of Archaeology at Simon Fraser University. To reduce the likelihood of contamination and detect it if it did occur, strict contamination controls, including the analysis of blank extracts, were undertaken [29]. Permission to include the archaeological salmonid samples re-analyzed in this study was granted by the archaeologists who originally provided the samples to the Simon Fraser University Ancient DNA Laboratory.

### PCR amplification

PCR amplifications were performed on a Mastercycler Personal or Gradient thermal cycler (Eppendorf, Mississauga, ON, Canada) in a 25 or 30 μL reaction volume. The reaction volume for the *Clock1a*/*sdY* assays contained 1.5× PCR Gold Buffer (Applied Biosystems, Carlsbad, CA, USA), 2 mM MgCl_2_, 0.2 mM dNTP, 0.45 μM of each *sdY* primer, 0.3 μM of each *Clock1a* primer, BSA (1 mg/mL), 1–5 μL DNA solution, and 0.75–2.25 U AmpliTaq Gold (Applied Biosystems, Carlsbad, CA, USA). The reaction conditions for the D-loop/*sdY* assays were the same as above, except for the primer concentrations, which were as follows: 0.6 μM of each *sdY* primer, 0.1 μM of each D-loop primer. The thermal conditions of the PCRs consisted of an initial denaturation step at 95 °C for 12 min followed by 60 cycles at 95 °C for 30 s (denaturation), 54 °C for 30 s (annealing), and 72 °C for 40 s (extension), and a final extension step at 72 °C for 7 min. Negative PCR controls were included in each PCR setup to monitor for contamination. The negative controls amplified alongside the dilution series included 2.5 μL of the distilled H_2_O used to prepare the dilutions.

### Sex identification

Five microliters of PCR product were pre-stained with SYBR Green I (Life Technologies, Carlsbad, CA, USA), electrophoresed on a 2% or 3% agarose gel, and visualized with a Dark Reader transilluminator (Clare Chemical Research, Dolores, CO, USA). Due to their similar size (~13 bp difference), the separation of the fragments targeted by the *Clock1a*/*sdY* assays could not be sufficiently resolved using a 2% agarose gel. Consequently, a higher percentage agarose gel (3% agarose) run at 100 v for 99 min was used to separate the fragments amplified by the *Clock1a*/*sdY* assay. PCR products generated by the D-loop/*sdY* assays were typically separated using a 2% agarose gel run at 100 V for 30 to 60 min. The size and intensity of the PCR products generated by the assays was evaluated by visually inspecting the electrophoresis gels. A sample was confidently identified as a male if *sdY* and the IPC or just *sdY* was amplified with both assays (Table 4). Samples were identified as female if *sdY* failed to amplify and both IPCs were amplified (Table 4). As a quality assurance measure, a sex identity was not assigned to a sample if the assays yielded inconsistent results or did not yield amplified DNA (Table 4).

**Table 4:** Potential results of the two assays and the final sex identification that would be assigned to a sample in each of these scenarios.

Assay	Marker	Potential Scenarios														
		1	2	3	4	5	6	7	8	9	10	11	12	13	14	15
*Clock1a*/*sdY*	*Clock1a*	+^1^	-	+	-	+	-	-	+	+	-	-	-	+	+	+
	*sdY*	+	+	+	+	-	-	-	+	+	+	+	-	-	-	-
D-loop/*sdY*	D-loop	+	-	-	+	+	-	+	-	+	+	-	-	-	+	-
	*sdY*	+	+	+	+	-	-	-	-	-	-	-	+	-	+	+
**Final Sex ID**		♂^2^	♂	♂	♂	♀	N	N	N	N	N	N	N	N	N	N

^1^+ = Amplicon present, - = Amplicon not present

^2^♂ = Male, ♀ = Female, N = No sex identity assigned

### Sequence analysis and species identification

To confirm their species identities, we directly sequenced the D-loop fragments amplified from a subset of the archaeological samples with the D-loop/*sdY* assay. Since the archaeological remains have all been previously identified to the species-level through mtDNA analysis, we only sequenced the D-loop amplicons obtained from 12 samples to assess species identification accuracy. These samples included at least one sample from each of the six species and three archaeological sites represented in the set of analyzed archaeological samples. D-loop amplicons were directly sequenced with the amplification primers in the forward and/or reverse direction at the Eurofins Genomics sequencing facility (Toronto, ON). The obtained sequences were visually edited, truncated to remove the primer sequences, and assembled using ChromasPro (http://www.technelysium.com.au). The resulting edited sequences were compared to reference sequences in GenBank through a BLAST search [30]. In BioEdit [31], the edited sequences were aligned with salmonid reference sequences using ClustalW [32] and trimmed to the same length. Species identifications were then assigned to the samples using the procedure described by Yang et al. [19].

## Results

### Modern samples

**Fig 1:**
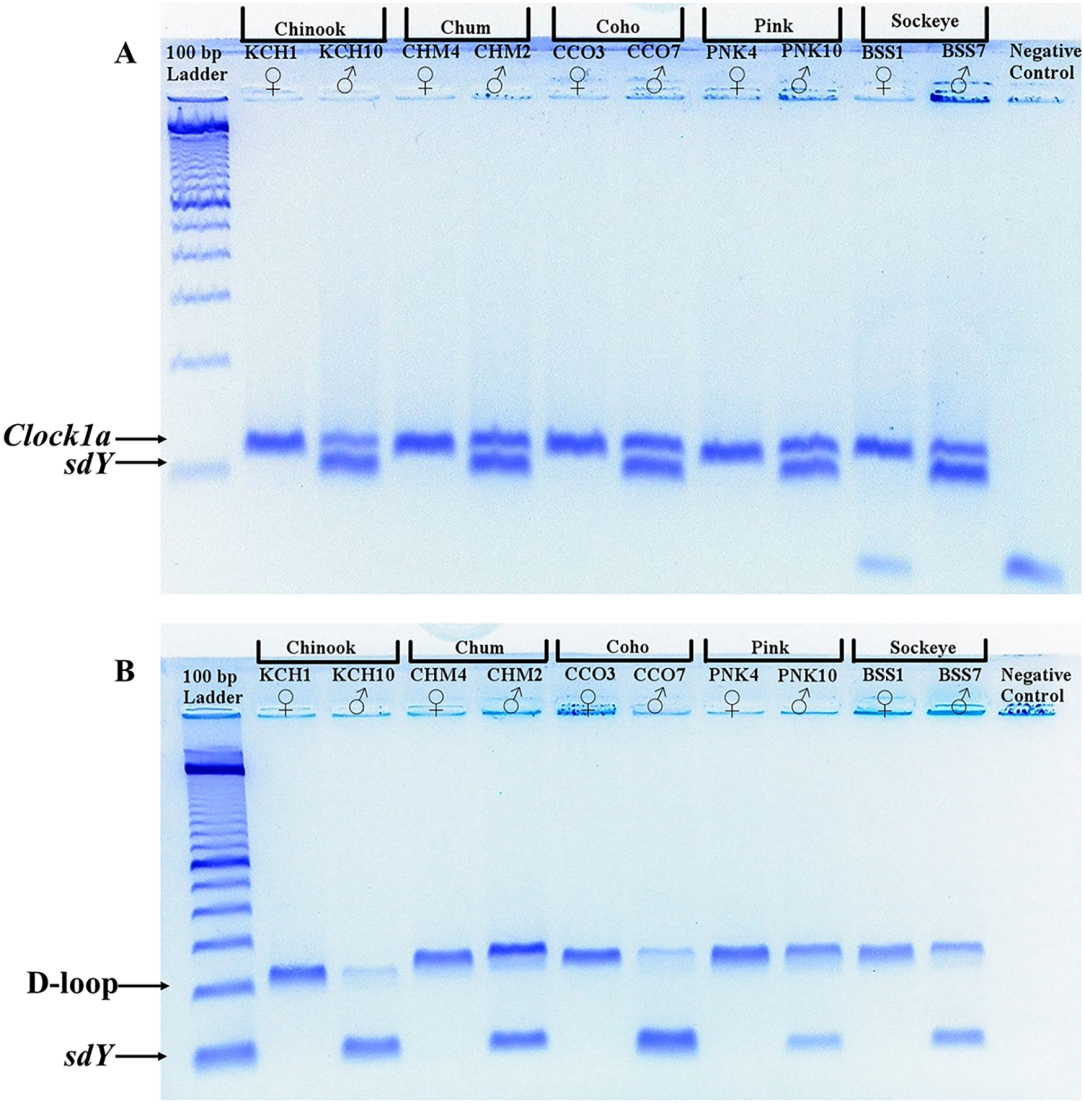
Negative images of electrophoresis gels showing the (A) *Clock1a*/*sdY* and (B) D-loop/*sdY* PCR assay results for modern male and female samples from five Pacific salmonid species. The approximate location of the IPC and *sdY* amplicons are indicated by the labelled arrows. The 100 bp ladder used to estimate the size of the amplicons is from Invitrogen (Waltham, MA, USA).

DNA was amplified from all of the modern salmonid samples with both the *sdY*/D-loop and *sdY*/*Clock1a* assays (Fig 1). The sex identifications assigned to each of the modern samples with both assays were concordant, thereby allowing a final consensus sex identity to be assigned to each of the modern samples (Fig 1 and S1 Table). The final consensus sex identities assigned to each of the modern samples matched their known phenotypic sex (S1 Table). None of the negative PCR controls associated with the modern samples yielded PCR products of the expected size.

### Dilution series

The IPCs were successfully amplified with both assays from the 10-, 100-, 1,000-, 10,000-, and 100,000-fold dilutions of the female Chinook salmon sample (KCH4) (S1 Fig). Similarly, *sdY* and the IPCs were amplified with both assays from the 10- to 100,000-fold dilutions of the male Chinook salmon sample (KCH9) (S1 Fig). No DNA was amplified from the negative PCR controls or the 1,000,000–fold dilution of either KCH4 or KCH9 (S1 Fig), with one exception. The D-loop fragment was weakly amplified from the 1,000,000-fold dilution of KCH9 (S1 Fig). Accordingly, the highest dilution of each sample that could be assigned a sex identity was the 100,000-fold dilution. The concentration of DNA in the 100,000-fold dilutions of KCH4 and KCH9 is estimated to be approximately 15.6 pg/μL (KCH4) and 15.8 pg/μL (KCH9). The reaction volume for the 100,000-fold dilution of KCH4 contained approximately 39.0 pg of DNA, whereas the 100,000-fold dilution of KCH4 contained approximately 39.4 pg of DNA.

### Archaeological samples

**Fig 2:**
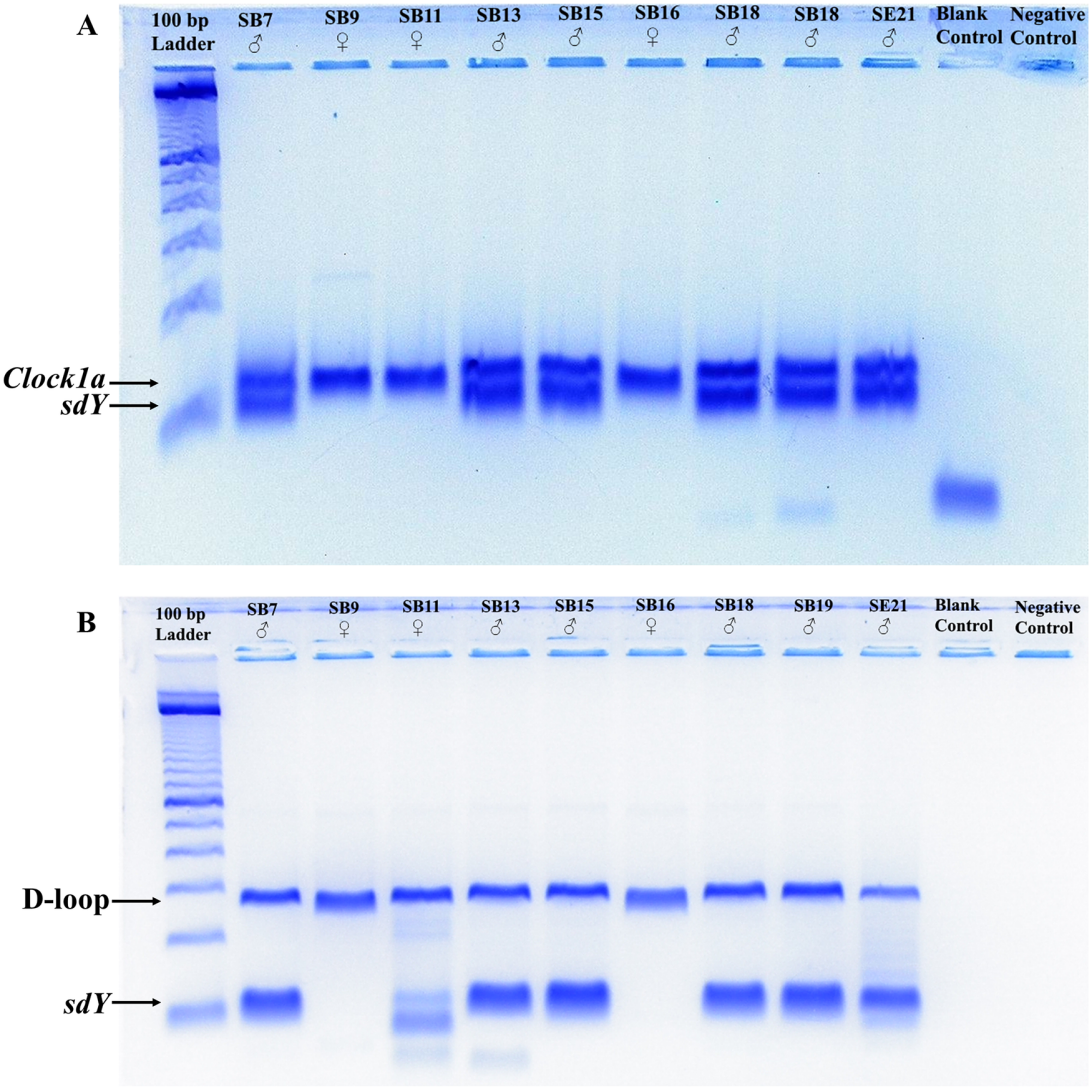
Negative images of electrophoresis gels showing the (A) *Clock1a*/*sdY* (B) D-loop/*sdY* assay results for nine of the analyzed archaeological salmonid samples. The approximate location of the IPC and *sdY* amplicons are indicated by the labelled arrows. The 100 bp ladder used to estimate the size of the amplicons is from Invitrogen (Waltham, MA, USA). Note: For SB11, the D-loop/*sdY* assay (B) produced two weak nonspecific bands only slightly smaller than the predicted size of the *sdY* amplicon, suggesting they might represent *sdY*. However, the *Clock1a*/*sdY* assay (A) only yielded a fragment of *Clock1a*, confirming the nonspecific bands likely do not represent *sdY*, verifying SB11’s female identity.

Among the 75 archaeological samples that were tested, only two samples (SA2 and SD23) consistently failed to yield DNA, and therefore could not be assigned a sex (S2 Table). Of the 73 archaeological samples that yielded DNA, 70 samples yielded sex identification results that were consistent across both assays and could therefore be assigned a final consensus sex identity (Fig 2 and S2 Table). At least two samples from each of the species represented among the archaeological remains were successfully assigned a sex identification (Table 5 and S2 Table). In total, 37 of these samples were identified as male (53%) and 33 were identified as female (47%) (Table 5 and S2 Table). Table 5 presents the overall sex ratio for each site and the sex ratios for each of the salmonid species represented at the sites. At each of the sites, both the overall sex ratio and the sex ratios for each of the identified species were not significantly male or female biased (Exact binomial test, two-tailed, all p>0.05). Overall sex ratios also did not significantly differ among sites (Fisher’s exact test, two-tailed, p = 0.17).

**Table 5:** Sex ratios (Number of identified males to females) by archaeological site and species.

Site	Chinook	Chum	Coho	Pink	Rainbow/Steelhead Trout	Sockeye	Overall
Kawumkam Springs Midden	1:1	—	—	—	6:1	—	7:2
Keatley Creek	3:4	—	1:1	—	—	19:22	23:27
Say-Umiton	—	5:4	—	2:0	—	—	7:4

Three archaeological samples (SD24, SE35, and SE40) yielded DNA but were not assigned a final consensus sex identity on account of the assays yielding inconsistent results (S2 Table). D-loop was repeatedly amplified from all three of these samples, but the amplification of nuclear DNA was variable. In the case of SE35, *sdY* was amplified once with both the D-loop/*sdY* and *Clock1a*/*sdY* assay, but could not be re-amplified with either assay. Similarly, Clock1a was amplified from SD24 and SE40, but failed to amplify at least two other times. These inconsistent results may reflect allelic dropout related to DNA degradation or rare sequence variations in the *Clock1a* and *sdY* genes. Amplicons approximating the expected size of the targeted products were not amplified from any of the negative PCR controls or the blank extraction controls.

### Species identification

D-loop sequences were successfully obtained from the 12 samples that underwent sequencing. BLAST searches indicated each of the samples’ D-loop sequences most closely resembled reference sequences from Pacific salmonid species. Through multiple alignment and phylogenetic analysis, we were able to assign a species-level identification to each of the sequenced samples (S2 Table). The species identities assigned to each of the samples matched their previously assigned species identities (S2 Table) [23,25,26].

## Discussion

### Authenticity of the ancient DNA results

Multiple lines of evidence indicate the sex identification results obtained for the archaeological salmonid samples are authentic and not the result of contamination. First, all pre-PCR laboratory work involving the archaeological samples was conducted in a dedicated aDNA laboratory that is physically separated from the modern DNA and post-PCR laboratories. Second, the archaeological samples were previously rigorously decontaminated using a combination of chemical washes and UV irradiation [23,25,26]. Third, no amplicons of the expected sized were amplified from any of the negative PCR or blank extraction controls in this or previous studies [23,25,26]. Fourth, the species identities assigned to all 12 of the samples whose D-loop amplicons were sequenced matched the species identities assigned to them in previous studies (S2 Table) [23,25,26].

### Sensitivity and cross-species applicability

In this study, we successfully assigned sex identities to 93% of the archaeological salmonid samples we analyzed with our method. The high proportion of samples that were successfully sexed highlights the high sensitivity of our method. However, as remains that did not previously yield mtDNA were not tested, our results likely overestimate the method’s sensitivity. Nonetheless, the sexing of nearly all of the remains previously identified to the species-level through mtDNA analysis, suggests our method’s sensitivity is comparable to the mtDNA-based assays [19,33] used to identify the remains. Furthermore, our successful sexing of 100,000-fold dilutions of modern male and female Chinook salmon samples, with estimated DNA concentrations of 15.6 and 15.8 pg/μl, further supports the sensitivity of our method. Assuming the C-value of the Chinook salmon genome is 2.45 pg [34], the successful sexing of these dilutions using only ~39 pg of total genomic DNA indicates our method works readily with only ~8 nuclear DNA templates.

Our results also indicate our sex identification method can be used to sex individuals from a number of Pacific salmonid species. The method’s cross-species applicability is demonstrated by our successful sex identification of modern samples from five salmon species, and archaeological samples from six species. We expect that the method can also be applied to other Pacific salmonids, such as cutthroat trout (*O*. *clarkii*), but additional tests are needed to confirm this possibility. The sensitivity and cross-species applicability exhibited by our sex identification method suggests it is an efficient means for sexing archaeological Pacific salmonid remains from a range of species.

### Reliability

In addition to being sensitive and having cross-species applicability, our proposed sex identification method has also proven to be able to produce reliable sex identities. The agreement between the sex we assigned to each of modern samples with our sex identification method, and their known phenotypic sex, highlights our method’s reliability. Our method’s reliability is partly due to its reliance on assays that screen for the presence of *sdY*, rather than other Y-linked markers (e.g., *GH-Y*) not as strongly associated with phenotypic sex [35,36]. Due to *sdY*’s critical role in controlling sex differentiation in Pacific salmonids, its presence or absence is a reliable proxy for phenotypic sex [17]. However, previous studies have identified modern Pacific salmonids with *sdY* genotypes inconsistent with their phenotypic sex [14,15,17], indicating our method may not always yield accurate sex identifications. Both *sdY*-positive females and *sdY*-negative males have been previously documented among modern Pacific salmonid populations [14,15,17]. Mutations and environmental factors, such as temperature and exposure to certain contaminants, may trigger sex reversals that result in individuals with discordant genotypic and phenotypic sexes [14]. Nevertheless, unless past conditions were more conducive to sex reversals, erroneous sex identifications caused by sex reversals will likely be minimal as less than 7% of contemporary Pacific salmonids have incongruent genotypic and phenotypic sexes [14–17].

The reliability of the sex identities assigned to archaeological samples with our method is enhanced by its reliance on two PCR assays, rather than a single assay, to sex samples. By using two assays, erroneous sex identifications or no calls caused by the dropout of *sdY* or the IPC due to DNA degradation can be detected [37]. As evidenced by the inconsistent sex identification results obtained for three of the archaeological samples (SD24, SE35, SE40), the dropout of single-copy nuclear markers, such as *sdY*, does occur when dealing with specimens with degraded DNA. The dropout of Y-linked markers is more common when the IPC used in a PCR assay outnumbers the Y-linked marker, as its higher copy number results in it outcompeting the Y-linked marker [38]. Our method is potentially susceptible to *sdY* dropout related to this issue as the IPCs in both the *Clock1a*/*sdY* and D-loop/*sdY* assays have higher copy numbers than *sdY*. However, we reduced the potential for *sdY* dropout and erroneous sex identifications caused by the IPCs outnumbering and outcompeting *sdY* by designing both assays to favor the amplification of *sdY* [18]. Both assays were designed to preferentially amplify *sdY* by skewing the primer ratio in favour of the *sdY* primers and targeting an *sdY* fragment shorter than the IPCs [18]. Among the male samples, the stronger amplifications obtained for *sdY* relative to those obtained for the IPC, particularly D-loop, indicates *sdY* was indeed preferentially amplified (Figs 1 and 2 and S1 Fig). In addition to the above factors, primer-template mismatches can also result in *sdY* dropout and erroneous sex identifications [38]. Although not addressed here, the likelihood of *sdY* dropout caused by primer-template mismatches could be lessened by using alternative *sdY* primers in one of the assays.

Although they can potentially contribute to *sdY* dropout and erroneous sex identifications, the IPCs included in both assays play a critical role in validating the sex identities assigned to archaeological samples. Among samples that did not yield *sdY* amplicons, the amplification of both IPCs indicates their lack of *sdY* likely reflects their female sex rather than degradation or inhibition [18]. Without the inclusion of an IPC, reliably identifying samples as female would be difficult as inhibited, degraded, and female samples would produce identical results: no amplicons.

### Detection of contamination and species identification

As archaeological samples are susceptible to contamination, embedding means of detecting contamination within aDNA analyses is of critical importance [29]. Our method’s use of two assays fulfills this requirement as the assays act as independent PCR replications that can aid in the detection of contamination and authentication of sex identification results. Furthermore, sequencing the various fragments amplified by the two assays provides an additional means for detecting contamination. The generation of conflicting taxonomic identities through the sequence analysis of different fragments from the same sample is suggestive of contamination [19,33]. Moreover, by sequencing the D-loop fragment targeted by the D-loop/*sdY* assay, a sample’s species identity can also be determined [19]. Through the sequence analysis of this D-loop fragment, we successfully assigned species-level identifications to 12 archaeological remains, a task that is typically not possible though morphological analyses [39]. Consequently, this method will allow researchers to simultaneously determine past salmonid fisheries’ species and sex preferences.

### Archaeological implications

The analyzed salmonid remains from each of the sites examined in this study were not meant to be representative samples, which limits our data’s interpretive potential. Nonetheless, our data allows some hypotheses to be drawn about the sex-selectivity of the pre-Contact salmonid fisheries in northwestern North America. The lack of a biased sex ratio among the relatively large sample of sexed salmonid remains from Keatley Creek suggests sex-selective salmon fishing was not a pervasive practice among the site’s inhabitants. Conversely, little can be said about the sex-selectivity of the KSM’s and Say-Umiton’ssalmonid fisheries given the likely unrepresentativeness of the small number of sexed salmonid remains (n = 9 and n = 11, respectively) from these sites. Furthermore, the curatorial history of the KSM assemblage, specifically the loss of an unknown number of remains between excavation and aDNA analysis, also makes our KSM sample’s representativeness questionable [21]. Consequently, establishing the sex-selectivity of pre-Contact salmonid fisheries in the Upper Klamath Basin will require examining remains from other sites in that region. In the case of Say-Umiton, establishing the sex-selectivity of its salmonid fishery will require analyzing additional remains from the site.

## Conclusion

In this study, we developed and optimized a highly-sensitive DNA-based method for the sex identification of archaeological Pacific salmonid remains. This method integrates two PCR assays that co-amplify an IPC (*Clock1a* or D-loop) and this genus’ master sex-determining gene: *sdY*. In summary,

1. Using this method, we successfully sexed 70 of the 75 (93%) mtDNA-identified archaeological Pacific salmonid samples we analyzed. This suggests the method has a high sensitivity comparable to that of mtDNA-based species identification assays, making it an efficient sex identification method for archaeological Pacific salmonid remains.
2. The sex identities assigned with this method to all 72 of the analyzed modern Pacific salmonid samples matched their known phenotypic sex, highlighting the method’s reliability. Reflecting the method’s sensitivity, dilutions of DNA samples from modern Chinook salmon could be assigned to the correct sex using as little as ~39 pg of total genomic DNA.
3. As evidenced by the successful sex identification of samples from six Pacific salmonid species (Chinook, chum, coho, pink, sockeye, and rainbow/steelhead trout), the method is applicable to remains from multiple Pacific salmonid species.
4. By sequencing the D-loop fragment used as an IPC in the D-loop/*sdY* assay, species-level identifications can be assigned to samples. This will enable the sex and species preferences of past salmonid fisheries to be determined in tandem.

Although we focused on salmonids from a single genus, our findings highlight the potential of using *sdY*-based assays to sex archaeological remains from other salmonids, such as Atlantic salmonids (*Salmo* spp.) and char (*Salvelinus* spp.), that share this master sex-determining gene [17]. More broadly, our results highlight the potential of using aDNA analysis to assign sex identities to archaeological fish remains from species whose sex is genetically determined. By enabling the sex identification of fish remains, aDNA analysis can shed light on the sex-selective fishing strategies employed by past peoples.

## Supporting information

S1 Table Species and phenotypic sex information and sex identification results for the modern Pacific salmonid samples analyzed in this study. (PDF)

S2 Table Sex and species identification results for the archaeological Pacific salmonid samples analyzed in this study. (PDF)

S1 Fig Negative images of electrophoresis gels showing the (A) *Clock1a*/*sdY* (B) D-loop/*sdY* PCR assay results for dilutions of a modern female (KCH4) and male (KCH9) Chinook salmon sample. The approximate location of the IPC and *sdY* amplicons are indicated by the labelled arrows. The 100 bp ladder used to estimate the size of the amplicons is from Invitrogen (Waltham, MA, USA).
(PDF)
