# Supplementary material for: An efficient and reliable DNA-based sex identification method for archaeological Pacific salmonid (Oncorhynchus spp.) remains
Source: PLoS One. 2018 Mar 14;13(3):e0193212. doi: 10.1371/journal.pone.0193212 (PMC5851554; doi:10.1371/journal.pone.0193212)
Supplement: S1 Table — (PDF) [file pone.0193212.s001.pdf]

S1 Table. Species and phenotypic sex information and sex identification results for the modern Pacific salmonid samples analyzed in this study.

Sample ID	Species	Phenotypic Sex	*Clock1a*/*sdY* Assay Sex ID	D-loop/*sdY* Assay Sex ID	Consensus Sex ID
BKS1	Sockeye	♀[1]	♀	♀	♀
BKS2	Sockeye	♀	♀	♀	♀
BKS3	Sockeye	♂	♂	♂	♂
BKS4	Sockeye	♂	♂	♂	♂
BSS1	Sockeye	♀	♀	♀	♀
BSS2	Sockeye	♂	♂	♂	♂
BSS3	Sockeye	♀	♀	♀	♀
BSS4	Sockeye	♂	♂	♂	♂
BSS5	Sockeye	♀	♀	♀	♀
BSS6	Sockeye	♂	♂	♂	♂
BSS7	Sockeye	♂	♂	♂	♂
CCO1	Coho	♀	♀	♀	♀
CCO2	Coho	♀	♀	♀	♀
CCO3	Coho	♀	♀	♀	♀
CCO4	Coho	♀	♀	♀	♀
CCO5	Coho	♀	♀	♀	♀
CCO6	Coho	♂	♂	♂	♂
CCO7	Coho	♂	♂	♂	♂
CCO8	Coho	♂	♂	♂	♂
CCO9	Coho	♂	♂	♂	♂
CCO10	Coho	♂	♂	♂	♂
CHC1	Chinook	♀	♀	♀	♀
CHC2	Chinook	♀	♀	♀	♀
CHC3	Chinook	♀	♀	♀	♀
CHC4	Chinook	♀	♀	♀	♀
CHC5	Chinook	♀	♀	♀	♀
CHC6	Chinook	♂	♂	♂	♂
CHC7	Chinook	♂	♂	♂	♂
CHC8	Chinook	♂	♂	♂	♂
CHC9	Chinook	♂	♂	♂	♂
CHC10	Chinook	♂	♂	♂	♂
CHM1	Chum	♀	♀	♀	♀

CHM2	Chum	♂	♂	♂	♂
CHM3	Chum	♂	♂	♂	♂
CHM4	Chum	♀	♀	♀	♀
CHM5	Chum	♂	♂	♂	♂
CRC1	Chum	♀	♀	♀	♀
CRC2	Chum	♀	♀	♀	♀
CRC3	Chum	♂	♂	♂	♂
CRC4	Chum	♂	♂	♂	♂
CRC5	Chum	♀	♀	♀	♀
CRC6	Chum	♂	♂	♂	♂
DCO1	Coho	♀	♀	♀	♀
DCO2	Coho	♀	♀	♀	♀
DCO3	Coho	♀	♀	♀	♀
DCO4	Coho	♀	♀	♀	♀
DCO5	Coho	♀	♀	♀	♀
DCO6	Coho	♂	♂	♂	♂
DCO7	Coho	♂	♂	♂	♂
DCO8	Coho	♂	♂	♂	♂
DCO9	Coho	♂	♂	♂	♂
DCO10	Coho	♂	♂	♂	♂
KCH1	Chinook	♀	♀	♀	♀
KCH2	Chinook	♀	♀	♀	♀
KCH3	Chinook	♀	♀	♀	♀
KCH4	Chinook	♀	♀	♀	♀
KCH5	Chinook	♀	♀	♀	♀
KCH6	Chinook	♂	♂	♂	♂
KCH7	Chinook	♂	♂	♂	♂
KCH8	Chinook	♂	♂	♂	♂
KCH9	Chinook	♂	♂	♂	♂
KCH10	Chinook	♂	♂	♂	♂
PNK1	Pink	♂	♂	♂	♂
PNK2	Pink	♂	♂	♂	♂
PNK3	Pink	♂	♂	♂	♂
PNK4	Pink	♀	♀	♀	♀
PNK5	Pink	♂	♂	♂	♂
PNK6	Pink	♂	♂	♂	♂

PNK7	Pink	♂	♂	♂	♂
PNK8	Pink	♀	♀	♀	♀
PNK9	Pink	♀	♀	♀	♀
PNK10	Pink	♂	♂	♂	♂

[1]♂ = Male, ♀ = Female
