# Supplementary material for: An efficient and reliable DNA-based sex identification method for archaeological Pacific salmonid (Oncorhynchus spp.) remains
Source: PLoS One. 2018 Mar 14;13(3):e0193212. doi: 10.1371/journal.pone.0193212 (PMC5851554; doi:10.1371/journal.pone.0193212)
Supplement: S2 Table — (PDF) [file pone.0193212.s002.pdf]

S2 Table. Sex and species identification results for the archaeological Pacific salmonid samples analyzed in this study.

Sample ID	Site	Previous Species ID	Repeat Species ID	*Clock1a*/*sdY* Assay Sex ID	D-loop/*sdY* Assay Sex ID	Consensus Sex ID
SA2	KC[1]	Chinook	—	PCR Failure	PCR Failure	N[2]
SA4	KC	Sockeye	Sockeye	♂	♂	♂
SA5	KC	Sockeye	—	♀	♀	♀
SA6	KC	Sockeye	—	♂	♂	♂
SA7	KC	Sockeye	Sockeye	♀	♀	♀
SA8	KC	Sockeye	—	♀	♀	♀
SA9	KC	Chinook	—	♂	♂	♂
SA10	KC	Chinook	—	♂	♂	♂
SA11	KC	Sockeye	—	♂	♂	♂
SA12	KC	Chinook	—	♀	♀	♀
SB1	KC	Chinook	Chinook	♂	♂	♂
SB3	KC	Sockeye	—	♀	♀	♀
SB5	KC	Sockeye	—	♀	♀	♀
SB7	KC	Sockeye	—	♂	♂	♂
SB9	KC	Sockeye	—	♀	♀	♀
SB11	KC	Sockeye	—	♀	♀	♀
SB13	KC	Sockeye	—	♂	♂	♂
SB15	KC	Sockeye	—	♂	♂	♂
SB16	KC	Sockeye	—	♀	♀	♀
SB18	KC	Sockeye	—	♂	♂	♂
SB19	KC	Sockeye	—	♂	♂	♂
SBC29	KSM	Rainbow/ Steelhead Trout	—	♂	♂	♂
SBC30	KSM	Rainbow/ Steelhead Trout	Rainbow/ Steelhead Trout	♀	♀	♀
SBC31	KSM	Rainbow/ Steelhead Trout	Rainbow/ Steelhead Trout	♂	♂	♂
SBC32	KSM	Rainbow/ Steelhead Trout	—	♂	♂	♂
SBC33	KSM	Chinook	—	♀	♀	♀

SBC35	KSM	Rainbow/ Steelhead Trout	—	♂	♂	♂
SBC36	KSM	Chinook	—	♂	♂	♂
SBC54	KSM	Rainbow/ Steelhead Trout	—	♂	♂	♂
SBC55	KSM	Rainbow/ Steelhead Trout	—	♂	♂	♂
SD6	KC	Sockeye	—	♂	♂	♂
SD9	KC	Sockeye	—	♀	♀	♀
SD13	KC	Sockeye	—	♂	♂	♂
SD17	KC	Sockeye	—	♀	♀	♀
SD20	KC	Sockeye	—	♂	♂	♂
SD22	KC	Sockeye	—	♂	♂	♂
SD23	KC	Sockeye	—	PCR Failure	PCR Failure	N
SD24	KC	Sockeye	—	PCR Failure/♀	♀	N
SD25	KC	Sockeye	—	♂	♂	♂
SD32	KC	Sockeye	—	♂	♂	♂
SD57	KC	Sockeye	—	♀	♀	♀
SD66	KC	Chinook	—	♀	♀	♀
SD68	KC	Chinook	—	♀	♀	♀
SD70	KC	Sockeye	—	♂	♂	♂
SD76	KC	Sockeye	—	♀	♀	♀
SD77	KC	Sockeye	—	♀	♀	♀
SD78	KC	Sockeye	—	♀	♀	♀
SD79	KC	Sockeye	—	♀	♀	♀
SD80	KC	Sockeye	—	♂	♂	♂
SE1	KC	Sockeye	—	♀	♀	♀
SE9	KC	Sockeye	—	♂	♂	♂
SE15	KC	Sockeye	—	♀	♀	♀
SE21	KC	Sockeye	—	♂	♂	♂
SE23	KC	Sockeye	—	♀	♀	♀
SE25	KC	Sockeye	—	♀	♀	♀
SE35	KC	Sockeye	—	PCR Failure/♂	♀/♂	N
SE40	KC	Sockeye	—	PCR Failure/♀	♀	N
SE45	KC	Sockeye	—	♂	♂	♂

SE47	KC	Sockeye	—	♀	♀	♀
SE48	KC	Chinook	Chinook	♀	♀	♀
SE49	KC	Coho	Coho	♂	♂	♂
SE50	KC	Sockeye	—	♀	♀	♀
SE51	KC	Sockeye	—	♀	♀	♀
SE52	KC	Coho	Coho	♀	♀	♀
ST4	SU	Chum	—	♂	♂	♂
ST7	SU	Chum	Chum	♀	♀	♀
ST10	SU	Chum	—	♂	♂	♂
ST18	SU	Chum	—	♀	♀	♀
ST24	SU	Chum	—	♂	♂	♂
ST28	SU	Chum	—	♂	♂	♂
ST106	SU	Chum	—	♂	♂	♂
ST244	SU	Pink	Pink	♂	♂	♂
ST291	SU	Chum	—	♀	♀	♀
ST531	SU	Pink	—	♂	♂	♂
ST560	SU	Chum	Chum	♀	♀	♀

[1]KC = Keatley Creek (EeRl-7), KSM = Kawumkan Springs Midden (35KL9-12), SU = Say-Umiton (DhHr-18)
[2]♂ = Male, ♀ = Female, N = No sex identity assigned
