# Supplementary material for: An efficient and reliable DNA-based sex identification method for archaeological Pacific salmonid (Oncorhynchus spp.) remains
Source: PLoS One. 2018 Mar 14;13(3):e0193212. doi: 10.1371/journal.pone.0193212 (PMC5851554; doi:10.1371/journal.pone.0193212)
Supplement: S1 Fig — The approximate location of the IPC and sdY amplicons are indicated by the labelled arrows. The 100 bp ladder used to estimate the size of the amplicons is from Invitrogen (Waltham, MA, USA). (PDF) [file pone.0193212.s003.pdf]

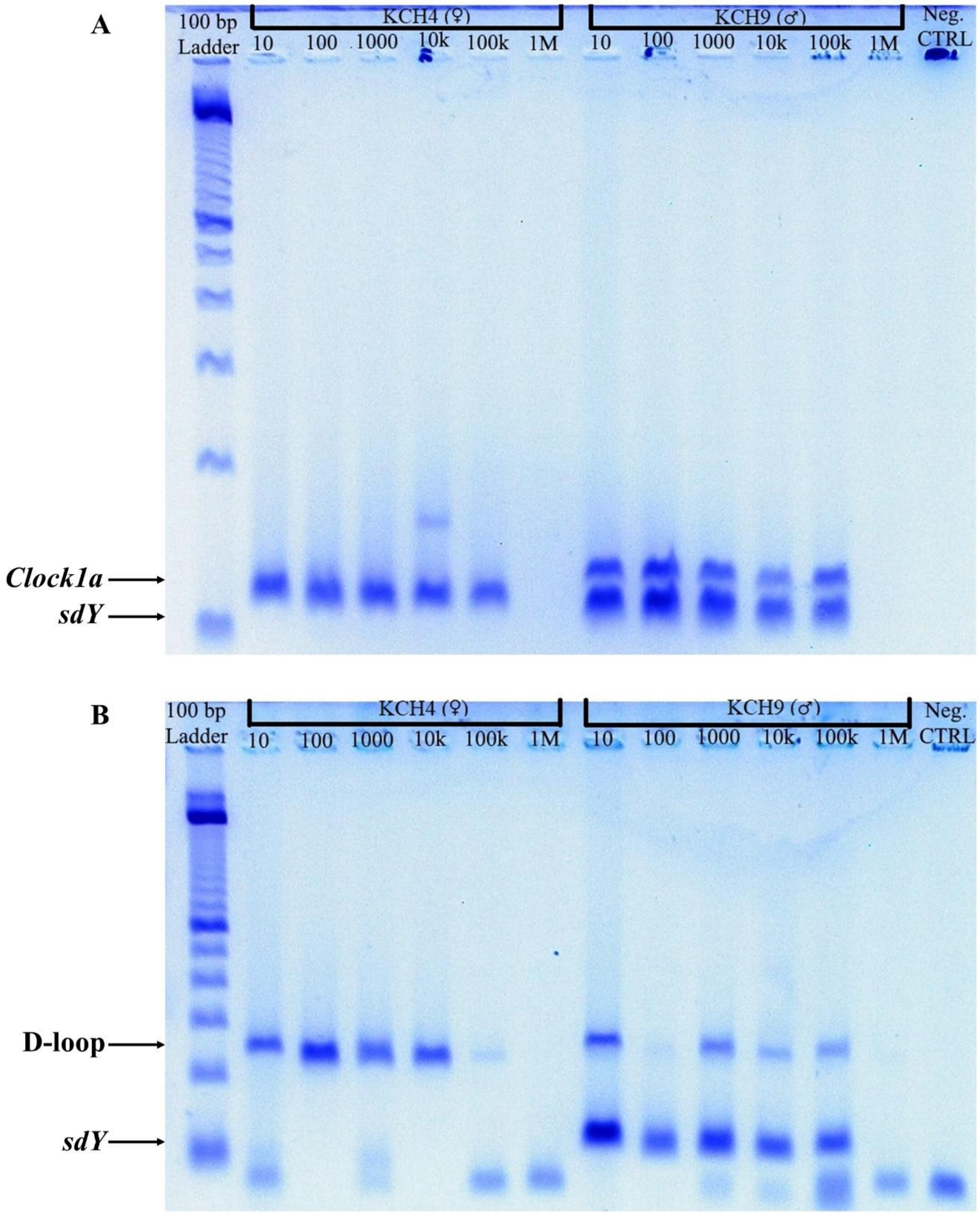

S1 Fig. Negative images of electrophoresis gels showing the (A) *Clock1a*/*sdY* (B) D-loop/*sdY* PCR assay results for dilutions of a modern female (KCH4) and male (KCH9) Chinook salmon sample.

The approximate location of the IPC and *sdY* amplicons are indicated by the labelled arrows. The 100 bp ladder used to estimate the size of the amplicons is from Invitrogen (Waltham, MA, USA).
